# Dichlorido{*N*-[2-(diphenyl­phosphan­yl)benzyl­idene]isopropyl­amine-κ^2^ *N*,*P*}palladium(II) dimethyl sulfoxide monosolvate

**DOI:** 10.1107/S1600536811013936

**Published:** 2011-04-16

**Authors:** Haleden Chiririwa, Reinout Meijboom, Bernard Omondi

**Affiliations:** a Department of Chemistry, University of Cape Town, Private Bag, Rondebosch, 7707, South Africa; b Research Centre for Synthesis and Catalysis, Department of Chemistry, University of Johannesburg, PO Box 524 Auckland Park, Johannesburg, 2006, South Africa

## Abstract

In the title Pd^II^ complex, [PdCl_2_(C_22_H_22_NP)]·(CH_3_)_2_SO, the Pd^II^ atom is coordinated in an NPCl_2_ coordination sphere by the N(imino) and P(phosphane) atoms of the ligand and by two Cl^−^ ions in a slightly distorted square-planar geometry [r.m.s. deviation = 0.081 (3) Å, plane defined by the four atoms around the Pd atom]. The dimethyl sulfoxide solvent mol­ecules form centrosymmetric dimers due to an inter­molecular C—H⋯O inter­action. The crystal structure is further stabilized through two inter­molecular C—H⋯π inter­actions.

## Related literature

For structures with related ligands, see: Ghilardi *et al.* (1992 ▶); Sanchez *et al.* (1998 ▶, 2001 ▶).
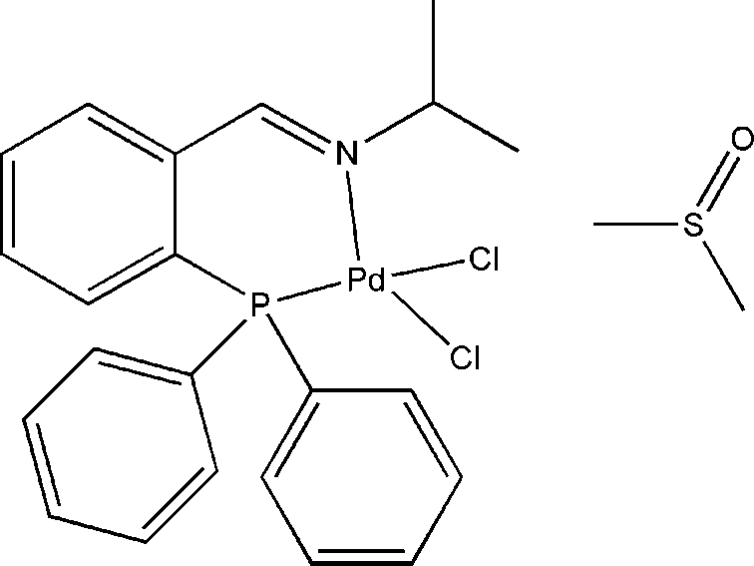


## Experimental

### 

#### Crystal data

[PdCl_2_(C_22_H_22_NP)]·C_2_H_6_OS
*M* *_r_* = 586.8
Triclinic, 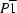

*a* = 8.9935 (2) Å
*b* = 10.0413 (2) Å
*c* = 13.9439 (3) Å
α = 91.189 (1)°
β = 97.957 (1)°
γ = 94.869 (1)°
*V* = 1241.93 (5) Å^3^
*Z* = 2
Mo *K*α radiation
μ = 1.13 mm^−1^
*T* = 173 K
0.20 × 0.10 × 0.05 mm

#### Data collection

Nonius KappaCCD diffractometer
Absorption correction: multi-scan (*XPREP*; Sheldrick, 2008 ▶) *T*_min_ = 0.806, *T*_max_ = 0.946
45019 measured reflections
6342 independent reflections
5437 reflections with *I* > 2σ(*I*)
*R*_int_ = 0.057

#### Refinement

*R*[*F* ^2^ > 2σ(*F* ^2^)] = 0.029
*wR*(*F* ^2^) = 0.069
*S* = 1.04
6342 reflections
284 parameters
H-atom parameters constrained
Δρ_max_ = 0.74 e Å^−3^
Δρ_min_ = −0.59 e Å^−3^

### 

Data collection: *COLLECT* (Nonius, 1998 ▶); cell refinement: *DENZO-SMN* (Otwinowski & Minor, 1997 ▶); data reduction: *DENZO-SMN*; program(s) used to solve structure: *SHELXS97* (Sheldrick, 2008 ▶); program(s) used to refine structure: *SHELXL97* (Sheldrick, 2008 ▶); molecular graphics: *DIAMOND* (Brandenburg & Putz, 2005 ▶) and *ORTEP-3* (Farrugia, 1997 ▶); software used to prepare material for publication: *WinGX* (Farrugia, 1999 ▶).

## Supplementary Material

Crystal structure: contains datablocks global, I. DOI: 10.1107/S1600536811013936/go2010sup1.cif

Structure factors: contains datablocks I. DOI: 10.1107/S1600536811013936/go2010Isup2.hkl

Additional supplementary materials:  crystallographic information; 3D view; checkCIF report

## Figures and Tables

**Table 1:** Selected bond lengths (Å)

Pd1—N24	2.0725 (17)
Pd1—P4	2.2188 (5)
Pd1—Cl3	2.2826 (5)
Pd1—Cl2	2.3838 (5)

**Table 2:** Hydrogen-bond geometry (Å, °) *Cg*3 and *Cg*4 are the centroids of the C11–C16 and C17–C22 rings, respectively.

*D*—H⋯*A*	*D*—H	H⋯*A*	*D*⋯*A*	*D*—H⋯*A*
C31—H31*A*⋯O29^i^	0.98	2.50	3.481 (4)	174
C10—H10⋯*Cg*3^ii^	0.95	2.84	3.643 (2)	146
C8—H8⋯*Cg*4^iii^	0.95	2.74	3.577 (3)	147

Symmetry codes: (i) 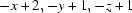; (ii) 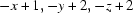; (iii) 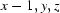.

**Fig. 1.**
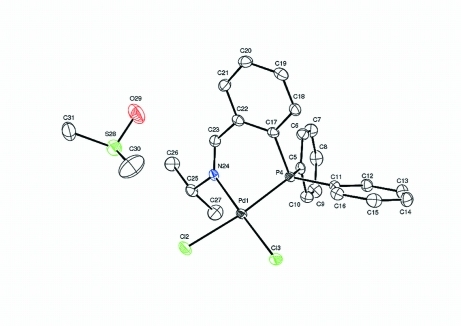
View of (I) (50% probability displacement ellipsoids).

**Fig. 2.**
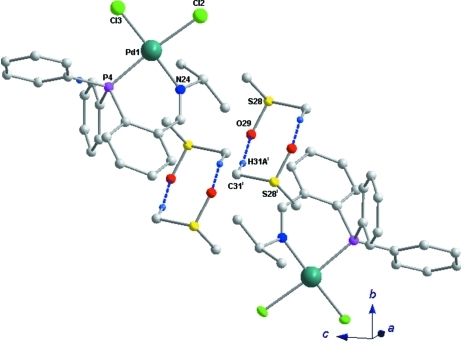
A perspective view of (I) showing intermolecular interactions between centrosymetric dimers of between solvent molecules [Symmetry operators: i = 2-x, 1-y, 1-z].

**Fig. 3.**
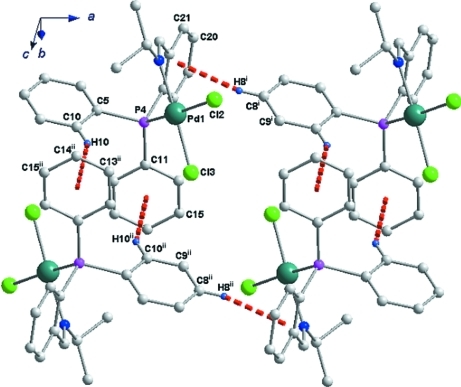
A perspective view of (I) showing molecules connected through C—H···π intermolecular interactions [Symmetry operators: i = x-1, y, z; ii = 1-x, 2-y, 2-z].

## supplementary crystallographic information

### Comment

In recent years, palladium complexes with iminophosphane ligands of the *N*-[(2-diphenylphosphanyl)benzylidene]amine type have been used as catalysts (or catalyst precursors) in a variety organic reactions. To the best of our knowledge, only a few structures have been determined so far, concerning the free ligand (2-diphenylphosphanyl-benzylidene)-isopropyl-amine, where the potentially bidentate ligand is chelated to the metal through the phosphorus and imino nitrogen atoms (Fig. 1). The title compound (I) has been synthesized earlier but there have been no reports of the crystal structure: the Pd^II^ center adopts a slightly distorted square planar geometry in with an r.m.s. deviation of 0.081 (3) Å from the planar geometry. Selected bond lengths are given in table 1.

In the structure of (I) the (CH_3_)_2_SO molecules are connected through a weak C—H···O intermolecular interaction forming centrosymmetric dimers (Fig. 2). In addition, the crystal lattice is further stabilized through two C—H···π intermolecular interactions (Fig 3). C10—H10···π (Cg of C11 to C16 atoms ring) joins two of the complex molecules into centrosymmetric dimers. Those combined with the C8—H8···π (Cg of C17 to C22 atoms ring) interaction, make additional  rings composed of four complex molecules for further stabilization of the crystal lattice.

### Experimental

To a dry CH_2_Cl_2_ (10 ml) solution of the precursor [Pd(COD)Cl_2_] (0.095 g, 0.3 mmol) was added isopropylamine (0.018 g, 0.3 mmol) in CH_2_Cl_2_ (10 ml) solution, and the reaction was stirred at room temperature for 1 hr. The yellow solution was concentrated under reduced pressure to half volume and the addition of *ca* 10 ml hexane caused precipitation of the complex, which was filtered off, washed with Et_2_O and dried under vacuum for 4 hrs. Yellow crystals used in the X-ray diffraction studies were grown by slow evaporation of a solution of the compound in a CH_2_Cl_2/_(CH_3_)_2_SO (1:1) solution at room temperature.

### Refinement

The methyl, methine and aromatic H atoms were placed in geometrically idealized positions and constrained to ride on their parent atoms, with C—H = 0.95 Å for aromatic, C—H = 0.99 Å for ^i^Pr CH, C—H = 0.95 Å for CH and C—H = 0.98 for Me groups.

### Crystal data

**Table:**

[PdCl_2_(C_22_H_22_NP)]·C_2_H_6_OS	*Z* = 2
*M**_r_* = 586.8	*F*(000) = 596
Triclinic, *P*1	*D*_x_ = 1.569 Mg m^−^^3^
Hall symbol: -P 1	Mo *K*α radiation, λ = 0.71073 Å
*a* = 8.9935 (2) Å	Cell parameters from 45103 reflections
*b* = 10.0413 (2) Å	θ = 3.0–28.7°
*c* = 13.9439 (3) Å	µ = 1.13 mm^−^^1^
α = 91.189 (1)°	*T* = 173 K
β = 97.957 (1)°	Block, yellow
γ = 94.869 (1)°	0.2 × 0.1 × 0.05 mm
*V* = 1241.93 (5) Å^3^	

### Data collection

**Table:**

Nonius KappaCCD diffractometer	5437 reflections with *I* > 2σ(*I*)
graphite	*R*_int_ = 0.057
1.0° ω scans, 60s	θ_max_ = 28.7°, θ_min_ = 3.0°
Absorption correction: multi-scan (*XPREP*; Sheldrick, 2008)	*h* = −12→12
*T*_min_ = 0.806, *T*_max_ = 0.946	*k* = −13→13
45019 measured reflections	*l* = −18→18
6342 independent reflections	

### Refinement

**Table:**

Refinement on *F*^2^	Primary atom site location: structure-invariant direct methods
Least-squares matrix: full	Secondary atom site location: difference Fourier map
*R*[*F*^2^ > 2σ(*F*^2^)] = 0.029	Hydrogen site location: inferred from neighbouring sites
*wR*(*F*^2^) = 0.069	H-atom parameters constrained
*S* = 1.04	*w* = 1/[σ^2^(*F*_o_^2^) + (0.0269*P*)^2^ + 1.3328*P*] where *P* = (*F*_o_^2^ + 2*F*_c_^2^)/3
6342 reflections	(Δ/σ)_max_ = 0.001
284 parameters	Δρ_max_ = 0.74 e Å^−^^3^
0 restraints	Δρ_min_ = −0.59 e Å^−^^3^

### Special details

**Table:**

Experimental. The intensity data was collected on a Nonius Kappa CCD diffractometer using an exposure time of 60 sec/per frame.
Geometry. All e.s.d.'s (except the e.s.d. in the dihedral angle between two l.s. planes) are estimated using the full covariance matrix. The cell e.s.d.'s are taken into account individually in the estimation of e.s.d.'s in distances, angles and torsion angles; correlations between e.s.d.'s in cell parameters are only used when they are defined by crystal symmetry. An approximate (isotropic) treatment of cell e.s.d.'s is used for estimating e.s.d.'s involving l.s. planes.
Refinement. Refinement of *F*^2^ against ALL reflections. The weighted *R*-factor *wR* and goodness of fit *S* are based on *F*^2^, conventional *R*-factors *R* are based on *F*, with *F* set to zero for negative *F*^2^. The threshold expression of *F*^2^ > σ(*F*^2^) is used only for calculating *R*-factors(gt) *etc*. and is not relevant to the choice of reflections for refinement. *R*-factors based on *F*^2^ are statistically about twice as large as those based on *F*, and *R*- factors based on ALL data will be even larger. >>> The Following Model and Quality ALERTS were generated - (Acta-Mode) <<< Format: alert-number_ALERT_alert-type_alert-level text 910_ALERT_3_C Missing # of FCF Reflections Below Th(Min) ···.. 8 911_ALERT_3_C Missing # FCF Refl Between THmin & STh/*L*= 0.600 4 244_ALERT_4_C Low 'Solvent' *U*_eq_ as Compared to Neighbors for S28 912_ALERT_4_C Missing # of FCF Reflections Above STh/*L*= 0.600 42 Noted.

### Fractional atomic coordinates and isotropic or equivalent isotropic displacement parameters (Å^2^)

**Table:**

	*x*	*y*	*z*	*U*_iso_*/*U*_eq_	
Pd1	0.692464 (17)	0.906752 (15)	0.782015 (11)	0.01442 (5)	
Cl2	0.81013 (7)	1.03910 (5)	0.66893 (4)	0.02557 (12)	
Cl3	0.79546 (6)	1.05312 (5)	0.90562 (4)	0.02177 (11)	
P4	0.60198 (6)	0.76339 (5)	0.88308 (4)	0.01342 (10)	
C5	0.3985 (2)	0.7298 (2)	0.86142 (14)	0.0160 (4)	
C6	0.3297 (2)	0.6002 (2)	0.84559 (16)	0.0211 (4)	
H6	0.3894	0.5267	0.8445	0.025*	
C7	0.1734 (3)	0.5788 (2)	0.83137 (17)	0.0267 (5)	
H7	0.1263	0.4904	0.8208	0.032*	
C8	0.0863 (3)	0.6857 (3)	0.83255 (17)	0.0278 (5)	
H8	−0.0205	0.6705	0.822	0.033*	
C9	0.1537 (3)	0.8148 (3)	0.84904 (18)	0.0276 (5)	
H9	0.0934	0.8878	0.8506	0.033*	
C10	0.3099 (2)	0.8373 (2)	0.86331 (16)	0.0215 (4)	
H10	0.3564	0.9258	0.8744	0.026*	
C11	0.6521 (2)	0.7857 (2)	1.01311 (14)	0.0157 (4)	
C12	0.5403 (2)	0.7771 (2)	1.07331 (16)	0.0198 (4)	
H12	0.4373	0.761	1.0461	0.024*	
C13	0.5799 (3)	0.7923 (2)	1.17326 (17)	0.0253 (5)	
H13	0.5038	0.7868	1.2144	0.03*	
C14	0.7297 (3)	0.8155 (2)	1.21262 (16)	0.0248 (5)	
H14	0.7563	0.8256	1.2809	0.03*	
C15	0.8418 (3)	0.8241 (2)	1.15317 (17)	0.0232 (5)	
H15	0.9446	0.8395	1.1809	0.028*	
C16	0.8037 (2)	0.8101 (2)	1.05361 (16)	0.0199 (4)	
H16	0.8802	0.8172	1.0129	0.024*	
C17	0.6770 (2)	0.6088 (2)	0.84994 (15)	0.0159 (4)	
C18	0.7437 (2)	0.5266 (2)	0.91970 (16)	0.0192 (4)	
H18	0.7501	0.5509	0.9865	0.023*	
C19	0.8007 (2)	0.4098 (2)	0.89261 (17)	0.0223 (5)	
H19	0.8482	0.356	0.9409	0.027*	
C20	0.7890 (3)	0.3707 (2)	0.79583 (17)	0.0233 (5)	
H20	0.827	0.2899	0.7776	0.028*	
C21	0.7210 (2)	0.4511 (2)	0.72572 (17)	0.0215 (4)	
H21	0.7113	0.424	0.6593	0.026*	
C22	0.6667 (2)	0.5713 (2)	0.75165 (15)	0.0172 (4)	
C23	0.5949 (2)	0.6484 (2)	0.67202 (15)	0.0181 (4)	
H23	0.5485	0.5992	0.6152	0.022*	
N24	0.58919 (19)	0.77410 (17)	0.67214 (12)	0.0151 (3)	
C25	0.5053 (2)	0.8390 (2)	0.58773 (16)	0.0213 (4)	
H25	0.5792	0.9031	0.5612	0.026*	
C26	0.4340 (3)	0.7429 (2)	0.50560 (17)	0.0295 (5)	
H26A	0.5125	0.6957	0.4808	0.044*	
H26B	0.3821	0.7929	0.4534	0.044*	
H26C	0.3613	0.678	0.5295	0.044*	
C27	0.3888 (3)	0.9202 (3)	0.62503 (19)	0.0332 (6)	
H27A	0.3169	0.8605	0.6542	0.05*	
H27B	0.335	0.9658	0.5711	0.05*	
H27C	0.4395	0.9867	0.6739	0.05*	
S28	0.84672 (7)	0.69728 (7)	0.47504 (5)	0.03166 (14)	
O29	0.7727 (2)	0.5699 (2)	0.50672 (16)	0.0482 (5)	
C30	0.9867 (4)	0.7594 (4)	0.5719 (3)	0.0637 (10)	
H30A	0.9381	0.784	0.6277	0.096*	
H30B	1.0443	0.8383	0.5513	0.096*	
H30C	1.0547	0.6901	0.5902	0.096*	
C31	0.9725 (4)	0.6499 (3)	0.3950 (2)	0.0425 (7)	
H31A	1.0412	0.5889	0.4276	0.064*	
H31B	1.0309	0.7298	0.3765	0.064*	
H31C	0.9146	0.6048	0.3369	0.064*	

### Atomic displacement parameters (Å^2^)

**Table:**

	*U*^11^	*U*^22^	*U*^33^	*U*^12^	*U*^13^	*U*^23^
Pd1	0.01427 (8)	0.01327 (8)	0.01514 (8)	−0.00108 (5)	0.00121 (6)	0.00175 (5)
Cl2	0.0312 (3)	0.0234 (3)	0.0205 (3)	−0.0086 (2)	0.0039 (2)	0.0057 (2)
Cl3	0.0260 (3)	0.0174 (2)	0.0203 (3)	−0.0042 (2)	0.0015 (2)	−0.00139 (19)
P4	0.0126 (2)	0.0129 (2)	0.0144 (2)	−0.00071 (18)	0.00179 (19)	0.00119 (19)
C5	0.0129 (9)	0.0209 (10)	0.0138 (9)	−0.0019 (8)	0.0021 (7)	0.0012 (8)
C6	0.0201 (10)	0.0199 (10)	0.0230 (11)	−0.0014 (8)	0.0035 (9)	−0.0005 (9)
C7	0.0218 (11)	0.0273 (12)	0.0284 (12)	−0.0099 (9)	0.0018 (9)	−0.0030 (10)
C8	0.0139 (10)	0.0409 (14)	0.0271 (12)	−0.0034 (9)	0.0008 (9)	0.0019 (10)
C9	0.0191 (11)	0.0315 (12)	0.0330 (13)	0.0060 (9)	0.0036 (10)	0.0035 (10)
C10	0.0177 (10)	0.0204 (10)	0.0264 (11)	0.0007 (8)	0.0033 (9)	0.0019 (9)
C11	0.0177 (10)	0.0144 (9)	0.0150 (10)	0.0006 (8)	0.0025 (8)	0.0015 (7)
C12	0.0180 (10)	0.0211 (10)	0.0197 (10)	−0.0017 (8)	0.0028 (8)	0.0013 (8)
C13	0.0290 (12)	0.0274 (12)	0.0211 (11)	0.0008 (9)	0.0094 (9)	0.0019 (9)
C14	0.0329 (13)	0.0253 (11)	0.0156 (10)	0.0058 (10)	−0.0008 (9)	0.0017 (9)
C15	0.0209 (11)	0.0224 (11)	0.0242 (11)	0.0029 (9)	−0.0054 (9)	0.0024 (9)
C16	0.0185 (10)	0.0193 (10)	0.0220 (11)	0.0018 (8)	0.0029 (8)	0.0019 (8)
C17	0.0132 (9)	0.0139 (9)	0.0213 (10)	−0.0008 (7)	0.0053 (8)	0.0017 (8)
C18	0.0198 (10)	0.0192 (10)	0.0186 (10)	−0.0011 (8)	0.0048 (8)	0.0021 (8)
C19	0.0212 (11)	0.0187 (10)	0.0277 (12)	0.0034 (8)	0.0042 (9)	0.0073 (9)
C20	0.0230 (11)	0.0166 (10)	0.0322 (12)	0.0037 (8)	0.0096 (9)	0.0011 (9)
C21	0.0221 (11)	0.0197 (10)	0.0234 (11)	0.0002 (8)	0.0073 (9)	−0.0027 (8)
C22	0.0133 (9)	0.0164 (10)	0.0219 (11)	−0.0016 (7)	0.0038 (8)	0.0027 (8)
C23	0.0170 (10)	0.0195 (10)	0.0175 (10)	−0.0012 (8)	0.0028 (8)	−0.0006 (8)
N24	0.0143 (8)	0.0180 (8)	0.0124 (8)	−0.0014 (6)	0.0011 (6)	0.0029 (7)
C25	0.0230 (11)	0.0217 (11)	0.0174 (10)	0.0005 (9)	−0.0027 (8)	0.0044 (8)
C26	0.0326 (13)	0.0304 (13)	0.0219 (12)	0.0032 (10)	−0.0089 (10)	0.0006 (10)
C27	0.0346 (14)	0.0353 (14)	0.0294 (13)	0.0144 (11)	−0.0034 (11)	0.0033 (11)
S28	0.0291 (3)	0.0336 (3)	0.0343 (3)	0.0071 (3)	0.0082 (3)	0.0067 (3)
O29	0.0432 (12)	0.0504 (13)	0.0560 (13)	0.0020 (10)	0.0234 (10)	0.0197 (10)
C30	0.056 (2)	0.070 (2)	0.061 (2)	0.0167 (18)	−0.0100 (18)	−0.0245 (19)
C31	0.0501 (17)	0.0439 (16)	0.0398 (16)	0.0093 (14)	0.0240 (14)	0.0124 (13)

### Geometric parameters (Å, °)

**Table:**

Pd1—N24	2.0725 (17)	C18—C19	1.385 (3)
Pd1—P4	2.2188 (5)	C18—H18	0.95
Pd1—Cl3	2.2826 (5)	C19—C20	1.385 (3)
Pd1—Cl2	2.3838 (5)	C19—H19	0.95
P4—C11	1.811 (2)	C20—C21	1.390 (3)
P4—C5	1.814 (2)	C20—H20	0.95
P4—C17	1.820 (2)	C21—C22	1.399 (3)
C5—C6	1.392 (3)	C21—H21	0.95
C5—C10	1.397 (3)	C22—C23	1.473 (3)
C6—C7	1.390 (3)	C23—N24	1.268 (3)
C6—H6	0.95	C23—H23	0.95
C7—C8	1.383 (4)	N24—C25	1.500 (3)
C7—H7	0.95	C25—C26	1.516 (3)
C8—C9	1.385 (3)	C25—C27	1.520 (3)
C8—H8	0.95	C25—H25	1
C9—C10	1.389 (3)	C26—H26A	0.98
C9—H9	0.95	C26—H26B	0.98
C10—H10	0.95	C26—H26C	0.98
C11—C12	1.394 (3)	C27—H27A	0.98
C11—C16	1.401 (3)	C27—H27B	0.98
C12—C13	1.392 (3)	C27—H27C	0.98
C12—H12	0.95	S28—O29	1.495 (2)
C13—C14	1.381 (3)	S28—C30	1.777 (3)
C13—H13	0.95	S28—C31	1.781 (3)
C14—C15	1.390 (3)	C30—H30A	0.98
C14—H14	0.95	C30—H30B	0.98
C15—C16	1.384 (3)	C30—H30C	0.98
C15—H15	0.95	C31—H31A	0.98
C16—H16	0.95	C31—H31B	0.98
C17—C18	1.395 (3)	C31—H31C	0.98
C17—C22	1.402 (3)		
			
N24—Pd1—P4	86.13 (5)	C17—C18—H18	119.7
N24—Pd1—Cl3	177.29 (5)	C18—C19—C20	120.6 (2)
P4—Pd1—Cl3	92.308 (19)	C18—C19—H19	119.7
N24—Pd1—Cl2	91.07 (5)	C20—C19—H19	119.7
P4—Pd1—Cl2	172.60 (2)	C19—C20—C21	119.2 (2)
Cl3—Pd1—Cl2	90.74 (2)	C19—C20—H20	120.4
C11—P4—C5	106.03 (9)	C21—C20—H20	120.4
C11—P4—C17	106.11 (9)	C20—C21—C22	120.9 (2)
C5—P4—C17	105.72 (9)	C20—C21—H21	119.5
C11—P4—Pd1	121.52 (7)	C22—C21—H21	119.5
C5—P4—Pd1	113.83 (7)	C21—C22—C17	119.37 (19)
C17—P4—Pd1	102.26 (7)	C21—C22—C23	116.68 (19)
C6—C5—C10	119.75 (19)	C17—C22—C23	123.88 (19)
C6—C5—P4	121.68 (16)	N24—C23—C22	126.2 (2)
C10—C5—P4	118.55 (16)	N24—C23—H23	116.9
C7—C6—C5	119.8 (2)	C22—C23—H23	116.9
C7—C6—H6	120.1	C23—N24—C25	120.52 (18)
C5—C6—H6	120.1	C23—N24—Pd1	125.16 (15)
C8—C7—C6	120.2 (2)	C25—N24—Pd1	114.31 (13)
C8—C7—H7	119.9	N24—C25—C26	114.55 (18)
C6—C7—H7	119.9	N24—C25—C27	108.31 (18)
C7—C8—C9	120.4 (2)	C26—C25—C27	111.5 (2)
C7—C8—H8	119.8	N24—C25—H25	107.4
C9—C8—H8	119.8	C26—C25—H25	107.4
C8—C9—C10	119.8 (2)	C27—C25—H25	107.4
C8—C9—H9	120.1	C25—C26—H26A	109.5
C10—C9—H9	120.1	C25—C26—H26B	109.5
C9—C10—C5	120.0 (2)	H26A—C26—H26B	109.5
C9—C10—H10	120	C25—C26—H26C	109.5
C5—C10—H10	120	H26A—C26—H26C	109.5
C12—C11—C16	119.77 (19)	H26B—C26—H26C	109.5
C12—C11—P4	120.23 (16)	C25—C27—H27A	109.5
C16—C11—P4	119.99 (16)	C25—C27—H27B	109.5
C13—C12—C11	119.9 (2)	H27A—C27—H27B	109.5
C13—C12—H12	120	C25—C27—H27C	109.5
C11—C12—H12	120	H27A—C27—H27C	109.5
C14—C13—C12	120.0 (2)	H27B—C27—H27C	109.5
C14—C13—H13	120	O29—S28—C30	107.28 (18)
C12—C13—H13	120	O29—S28—C31	106.12 (13)
C13—C14—C15	120.5 (2)	C30—S28—C31	96.87 (17)
C13—C14—H14	119.7	S28—C30—H30A	109.5
C15—C14—H14	119.7	S28—C30—H30B	109.5
C16—C15—C14	120.0 (2)	H30A—C30—H30B	109.5
C16—C15—H15	120	S28—C30—H30C	109.5
C14—C15—H15	120	H30A—C30—H30C	109.5
C15—C16—C11	119.8 (2)	H30B—C30—H30C	109.5
C15—C16—H16	120.1	S28—C31—H31A	109.5
C11—C16—H16	120.1	S28—C31—H31B	109.5
C18—C17—C22	119.21 (19)	H31A—C31—H31B	109.5
C18—C17—P4	121.73 (16)	S28—C31—H31C	109.5
C22—C17—P4	119.06 (15)	H31A—C31—H31C	109.5
C19—C18—C17	120.6 (2)	H31B—C31—H31C	109.5
C19—C18—H18	119.7		
			
N24—Pd1—P4—C11	173.54 (9)	C12—C11—C16—C15	0.8 (3)
Cl3—Pd1—P4—C11	−8.68 (8)	P4—C11—C16—C15	−178.52 (16)
N24—Pd1—P4—C5	−57.78 (9)	C11—P4—C17—C18	5.77 (19)
Cl3—Pd1—P4—C5	120.00 (8)	C5—P4—C17—C18	−106.56 (18)
N24—Pd1—P4—C17	55.74 (8)	Pd1—P4—C17—C18	134.06 (16)
Cl3—Pd1—P4—C17	−126.48 (7)	C11—P4—C17—C22	−174.50 (16)
C11—P4—C5—C6	−99.04 (18)	C5—P4—C17—C22	73.16 (17)
C17—P4—C5—C6	13.3 (2)	Pd1—P4—C17—C22	−46.22 (17)
Pd1—P4—C5—C6	124.78 (16)	C22—C17—C18—C19	0.6 (3)
C11—P4—C5—C10	79.32 (18)	P4—C17—C18—C19	−179.64 (16)
C17—P4—C5—C10	−168.29 (17)	C17—C18—C19—C20	−1.7 (3)
Pd1—P4—C5—C10	−56.85 (18)	C18—C19—C20—C21	0.9 (3)
C10—C5—C6—C7	0.3 (3)	C19—C20—C21—C22	1.0 (3)
P4—C5—C6—C7	178.61 (17)	C20—C21—C22—C17	−2.0 (3)
C5—C6—C7—C8	0.3 (3)	C20—C21—C22—C23	−179.3 (2)
C6—C7—C8—C9	−0.8 (4)	C18—C17—C22—C21	1.2 (3)
C7—C8—C9—C10	0.8 (4)	P4—C17—C22—C21	−178.53 (15)
C8—C9—C10—C5	−0.3 (4)	C18—C17—C22—C23	178.23 (19)
C6—C5—C10—C9	−0.2 (3)	P4—C17—C22—C23	−1.5 (3)
P4—C5—C10—C9	−178.65 (18)	C21—C22—C23—N24	−152.1 (2)
C5—P4—C11—C12	−0.86 (19)	C17—C22—C23—N24	30.8 (3)
C17—P4—C11—C12	−112.97 (17)	C22—C23—N24—C25	−176.85 (19)
Pd1—P4—C11—C12	131.15 (15)	C22—C23—N24—Pd1	4.3 (3)
C5—P4—C11—C16	178.44 (16)	P4—Pd1—N24—C23	−45.47 (17)
C17—P4—C11—C16	66.32 (18)	Cl2—Pd1—N24—C23	127.68 (17)
Pd1—P4—C11—C16	−49.55 (19)	P4—Pd1—N24—C25	135.59 (13)
C16—C11—C12—C13	−0.3 (3)	Cl2—Pd1—N24—C25	−51.26 (13)
P4—C11—C12—C13	179.01 (17)	C23—N24—C25—C26	−1.0 (3)
C11—C12—C13—C14	−0.2 (3)	Pd1—N24—C25—C26	177.99 (16)
C12—C13—C14—C15	0.1 (4)	C23—N24—C25—C27	124.1 (2)
C13—C14—C15—C16	0.4 (3)	Pd1—N24—C25—C27	−56.9 (2)
C14—C15—C16—C11	−0.8 (3)		

### Hydrogen-bond geometry (Å, °)

**Table:**

Cg3 and Cg4 are the centroids ofthe C11-C16 and C17-C22 rings, respectively.

**Table:**

*D*—H···*A*	*D*—H	H···*A*	*D*···*A*	*D*—H···*A*
C31—H31A···O29^i^	0.98	2.50	3.481 (4)	174
C10—H10···Cg3^ii^	0.95	2.84	3.643 (2)	146
C8—H8···Cg4^iii^	0.95	2.74	3.577 (3)	147

Symmetry codes: (i) −*x*+2, −*y*+1, −*z*+1; (ii) −*x*+1, −*y*+2, −*z*+2; (iii) *x*−1, *y*, *z*.
